# Gallium-68 Labeled Iron Oxide Nanoparticles Coated with 2,3-Dicarboxypropane-1,1-diphosphonic Acid as a Potential PET/MR Imaging Agent: A Proof-of-Concept Study

**DOI:** 10.1155/2017/6951240

**Published:** 2017-12-28

**Authors:** Maria-Argyro Karageorgou, Sanja Vranješ-Djurić, Magdalena Radović, Anna Lyberopoulou, Bratislav Antić, Maritina Rouchota, Maria Gazouli, George Loudos, Stavros Xanthopoulos, Zili Sideratou, Dimosthenis Stamopoulos, Penelope Bouziotis, Charalampos Tsoukalas

**Affiliations:** ^1^Institute of Nuclear & Radiological Sciences & Technology, Energy & Safety, National Center for Scientific Research “Demokritos”, Aghia Paraskevi, 15310 Athens, Greece; ^2^Department of Solid State Physics, NKUA, Athens, Greece; ^3^“Vinča” Institute of Nuclear Sciences, University of Belgrade, Mike Petrovica Alasa 12-14, 11000 Belgrade, Serbia; ^4^Department of Basic Medical Science, Laboratory of Biology, School of Medicine, NKUA, Athens, Greece; ^5^Department of Medical Physics, School of Medicine, University of Patras, P.O. Box 132 73, 265 04 Rion, Greece; ^6^Department of Medical Instruments Technology, Technological Educational Institute of Athens, 28 Ag. Spyridonos Street, 12210 Egaleo, Greece; ^7^Institute of Nanoscience and Nanotechnology, National Center for Scientific Research “Demokritos”, Aghia Paraskevi, 15310 Athens, Greece

## Abstract

The aim of this study was to develop a dual-modality PET/MR imaging probe by radiolabeling iron oxide magnetic nanoparticles (IONPs), surface functionalized with water soluble stabilizer 2,3-dicarboxypropane-1,1-diphosphonic acid (DPD), with the positron emitter Gallium-68. Magnetite nanoparticles (Fe_3_O_4_ MNPs) were synthesized via coprecipitation method and were stabilized with DPD. The Fe_3_O_4_-DPD MNPs were characterized based on their structure, morphology, size, surface charge, and magnetic properties.* In vitro* cytotoxicity studies showed reduced toxicity in normal cells, compared to cancer cells. Fe_3_O_4_-DPD MNPs were successfully labeled with Gallium-68 at high radiochemical purity (>91%) and their stability in human serum and in PBS was demonstrated, along with their further characterization on size and magnetic properties. The* ex vivo* biodistribution studies in normal Swiss mice showed high uptake in the liver followed by spleen. The acquired PET images were in accordance with the* ex vivo* biodistribution results. Our findings indicate that ^68^Ga-Fe_3_O_4_-DPD MNPs could serve as an important diagnostic tool for biomedical imaging.

## 1. Introduction

Iron oxide magnetic nanoparticles (IONPs) have been subjected to a variety of biomedical applications due to their remarkable nanoscale physicochemical properties. Their small size, high surface to volume ratio and size-dependent [1] magnetic properties make them ideal candidates for clinical applications, including magnetic resonance imaging (MRI), in which they serve as T2-contrast enhancement agents [2–4], hyperthermia treatment of cancer [5–7], cell separation [8], tissue repair [9], and magnetic force guided drug delivery [10]. Magnetite (Fe_3_O_4_) and its oxidized form maghemite (Fe_2_O_3_) are the most common IONPs, which compose the core in a typical core-shell nanoparticle structure. Among the unique magnetic properties that IONPs demonstrate upon reducing their magnetic core size above a critical diameter (less than 20 nm for spherical IONPs), superparamagnetism is worth noting. IONPs lose their magnetization after the removal of the magnetic field and thus can be controllable. Additionally, their surface can be modified with biocompatible coatings via various methods [11, 12], rendering them biocompatible and applicable for biological systems. Thanks to the ease of surface functionalization, IONPs are provided with colloidal stability via steric and electrostatic interactions and high loading capacity via functional (i.e., hydroxyl, carboxyl, amino, and thiol) groups, in order to be bound with many active targeting molecules (such as antibodies, aptamers, and peptides) [13–16], drugs, and detection elements (i.e., radionuclides for molecular imaging, fluorescent molecules) according to demand in biomedical applications.

Colloidal stability of IONPs is of great importance to be considered in order to develop an effective imaging agent able to circulate in the bloodstream for as long as it takes to be accumulated in the target-organ. However, uncoated nanoparticles are prone to opsonization and tend to agglomerate* in vivo* (i.e., via Van der Waals, magnetic dipole-dipole, and hydrophobic interactions), leading to the formation of micrometer-sized particles, which mostly accumulate in the organs of the Reticuloendothelial System (RES), resulting in rapid sequestration from blood circulation. On the other hand, surface coating with appropriate organic and inorganic [17, 18] coatings provides IONPs with stealth properties, increased stability, and reduced cytotoxicity, consequently improving their potential use for* in vivo* applications [19].

The need for early cancer diagnosis has led scientists to a tremendous effort of developing noninvasive multimodal imaging agents able to detect abnormalities* in vivo* more accurately. As a consequence, the necessity of synthesizing dual-modality imaging agents, such as radiolabeled IONPs, has emerged in the last years in biomedical sciences, due to their ability to circumvent the limitations of a single imaging modality. In fact, conventional imaging modalities, namely, X-ray computed tomography (CT), optical imaging (OI), and magnetic resonance imaging (MRI), as well as positron emission (PET) and single-photon emission (SPECT) tomography, widely used in nuclear medicine, possess their own advantages and disadvantages. Particularly, PET (and/or SPECT) provides qualitative images with high sensitivity and specificity; however the anatomical information via this modality is diminished. On the contrary, MRI provides necessary anatomical information due to its high spatial resolution and contrast in soft tissue, but it exhibits relatively poor sensitivity [20]. This is the case why radiolabeled IONPs surpass conventional imaging modalities, since the combination of these two modalities (PET/MRI and/or SPECT/MRI) in a dual-modality agent has a synergistic result, ultimately exhibiting a much improved potential for biomedical imaging. Several papers have been reported concerning radiolabeled IONPs developed for PET/SPECT-MRI imaging combined with other biomedical applications like controlled drug delivery and hyperthermia treatment to achieve both diagnosis and therapy of cancer [21–24].

PET radionuclides such as Fluorine-18 and Carbon-11, commonly used in clinical practice, require time consuming and expensive facilities (i.e., on-site cyclotron) to be produced. On the other hand, radionuclides obtained from a generator-based system have received significant attention since they are inexpensive and easily available at any time for clinical use. Among them, Gallium-68 (^68^Ga:* T*_1/2_ = 68 min) is an attractive radioisotope for PET imaging due to its high proportion of positron decay (89%) of 1,9 Mev (maximum energy) and its availability from a ^68^Ge/^68^Ga generator permitting three elutions per day. Specifically, the long-lived radionuclide ^68^Ge (*T*_1/2_ = 270.8 days) decays via electron capture, providing the ^68^Ga radionuclide. It is worthy to be mentioned that few papers have been reported in the literature, concerning radiolabeling with ^68^Ga radionuclide of IONPs, synthesized in various sizes and shapes, to create dual-modality conjugates able to detect malignancies [25–28].

The imaging agent described in this work consists of Fe_3_O_4_ MNPs, surface functionalized with the water soluble stabilizer 2,3-dicarboxypropane-1,1-diphosphonic acid (DPD) and radiolabeled directly with ^68^Ga for PET imaging. The substance DPD was used because it is water soluble and biocompatible, providing the appropriate dispersing stability and minimizing the potential cytotoxicity of the naked Fe_3_O_4_ MNPs. Furthermore, as a tetradentate ligand (with two phosphonates and two carboxylate groups), DPD serves as an effective chelating agent, as has been shown for ^90^Y, leading to the formation of a highly stable conjugate [29].* In vitro* stability studies were performed with ^68^Ga-Fe_3_O_4_-DPD MNPs, to assess the stability of the complex in biological media, while* in vitro* cytotoxicity studies of the Fe_3_O_4_-DPD MNPs were performed to evaluate their potential toxicity in both normal and cancer cell lines. To assess the* in vivo* behavior of the ^68^Ga-Fe_3_O_4_-DPD MNPs* ex vivo* biodistribution studies and dynamic and cumulative imaging studies were performed in normal animal models.

## 2. Materials and Methods

### 2.1. Chemicals

2,3-Dicarboxypropane-1,1-diphosphonic acid (DPD) was synthesized at the Laboratory for Radioisotopes of the “Vinča” Institute of Nuclear Sciences, according to a previously reported procedure [30]. All other reagents and solvents used in these studies were obtained from commercial sources without further purification. Iron(II) sulfate hepta-hydrate (FeSO_4_ × 7H_2_O), iron(III) sulfate hydrate (Fe_2_(SO_4_)_3_ × H_2_O), sodium hydroxide (NaOH), and aqueous ammonia solution (25%) were obtained from Sigma-Aldrich. Purified deionized water was prepared by the Milli-Q system (Millipore Co., Billerica, MA, USA). A lower activity commercial ^68^Ge/^68^Ga generator was acquired from ITG Garching (Garching, Germany). 30% HCl Suprapur (Merck, Darmstadt, Germany), acetone (Sigma-Aldrich), 2,4-pentanedione 99% (Alfa Aesar, Karlsruhe, Germany), and 37% HCl (Riedel-de Haën) were commercially available and used as received. Human serum was acquired from Sigma-Aldrich (St. Louis, MO, USA). The MTT tetrazolium salt, 3-(4,5-dimethylthiazol-2-yl)-2,5-diphenyltetrazolium bromide, was acquired from Thermo Fisher Scientific (Cat. number M6494).

### 2.2. Equipment

Radioactivity of the ^68^GaCl_3_ eluent was measured using a dose calibrator (Capintec, Ramsey, NJ). Thin layer chromatography (TLC) silica gel 60 sheets (5 × 10 cm) were purchased from Merck (Dermstadt, Germany) and along with a Radio-TLC Scanner (Scan-Ram, LabLogic, Sheffield, UK) were used in the determination of radiolabeling yield/purity and* in vitro* stability studies. PD-10 columns (GE Healthcare), containing Sephadex G-25 resin, were used for the purification of radiolabeled samples. Water was deionized to 18 MΩ·cm using an easy-pure water filtration system (Barnstead International, Dubuque, Iowa). Gamma scintillation counter, Cobra II, Canberra, Packard, was used to measure the radioactivity of each organ and blood samples in* ex vivo* biodistribution studies. AXIOS-150/EX (Triton Hellas) dynamic light scattering (DLS) apparatus equipped with a 30 mW He-Ne laser emitting at 658 nm and an Avalanche photodiode detector at an angle of 90° was used for the determination of the size distributions of the particles. Atomic force microscopy (AFM) images were obtained by means of a scanning probe microscope [NT-MDT Solver PRO]. Magnetic measurements of liquid samples were performed by means of a SQUID magnetometer (5.5 T MPMS, Quantum Design). An ultrafast absorbance spectrophotometer (SPECTROstarNano, BMG LABTECH) for microplates and cuvettes was used in the MTT viability assay.

The imaging studies were performed on a custom-made trimodal system, incorporating a PET, a SPECT, and an X-ray subsystem [31]. For the current study the combination of PET coincidence imaging and X-rays was used. The dual head PET system is based on a pair of Position Sensitive PhotoMultiplier Tubes (PSPMTs), coupled to a 5 × 10 cm^2^ bismuth germanium oxide (BGO) scintillator array, with a pixel size of 2 × 2 × 5 mm^3^ and readout based on programmable ADCs and FPGA. The two heads are placed at an 80 mm distance and predefined acquisition parameters are a timing window of 16 ns and an energy window between 350 and 700 keV. The system average spatial resolution in coincidence mode is 3.5 mm, the peak sensitivity is 13483 cps/MBq, and the energy resolution is 30%. The X-ray system consists of an X-ray tube and a CMOS detector, separated by a distance of 30 cm. The minimum pixel size is equal to 0.1 mm and the active area is approximately 12 × 12 cm^2^.

### 2.3. Synthesis of Fe_3_O_4_-DPD MNPs

Magnetite nanoparticles were prepared by the alkali-mediated chemical coprecipitation of Fe^2+^ and Fe^3+^ ions (1 : 2 ratio), as described elsewhere [32]. In a typical experiment, ferrous sulfate heptahydrate (0.1 M) and ferric sulfate hydrate (0.2 M) were dissolved in deionized water. Subsequently 25% ammonia solution (ca. 20 ml) was injected into the flask and stirring was continued for 1 h at 50°C to allow the growth of the nanoparticles. The solution was subjected to magnetic decantation followed by repeated washing with distilled water. After the magnetite synthesis, the coating reaction was carried out by the addition of 0.25 g/2 mL DPD water solution (Fe_3_O_4_ : DPD = 1 : 1). The pH of the resultant mixture was adjusted to 8-9 by addition of 6 M NaOH. Then, the mixture was stirred overnight at room temperature and further subjected to dialysis against deionized water (MWCO 12 kDa) for 1 d to remove the excess of unreacted DPD.

### 2.4. Physicochemical Characterization of MNPs

The phase analysis of the synthesized MNPs powder was performed on a Philips PW1710 X-ray diffractometer. The data were collected in the angular range 10–50° (2*θ*) with a step size of 0.06° and a counting time of 50 s per step. High resolution transmission electron microscopy (HRTEM) was employed to characterize the morphology, size and size distribution of the MNPs. The samples of MNPs were prepared by placing one drop of a dilute suspension of MNPs in water on a carbon-coated copper grid and allowing the solvent to evaporate at room temperature. The surface charge of the MNPs was investigated through zeta potential measurements (Zetasizer Nano, Malvern instruments, UK) at pH between 1 and 11. Fourier transform-infrared (FTIR) measurements were carried out at room temperature on a Nicolet 380 spectrometer (Thermo Fischer Scientific, USA) in the spectral range 4000–400 cm^−1^, with 4 cm^−1^ resolution. Thermogravimetric analysis (TGA) was carried out with a SDT Q600 TGA/DSC instrument (TA Instruments) up to 850°C, by heating the sample under a nitrogen flow at a heating rate of 10°C/min. The residual weight accounts for the mass of iron oxide nanoparticles in the ferrofluid. Magnetic measurements of powder samples were performed on a SQUID magnetometer. Magnetization versus temperature, *M*(*T*), was measured in 2–300 K temperature range, under zero-field-cooled (ZFC) and field-cooled (FC) regimes, in 20 Oe. Hysteresis loops were measured at 300 K in ZFC regime.

### 2.5. *In Vitro* Cytotoxicity Study of Fe_3_O_4_-DPD MNPs

Two epithelial cell lines were used for the evaluation of cytotoxicity of Fe_3_O_4_-DPD MNPs, namely, HEK293T and 4T1. The normal cell line HEK293T is a highly transfectable derivative of human embryonic kidney 293 cell line that stably expresses the SV40 large T antigen. The 4T1 cell line is a highly breast metastatic tumorigenic 6-thioguanine resistant cell line derived from mouse that can metastasize to the lung, liver, lymph nodes, and brain, while the primary tumor is growing in situ. HEK293 are used as the control group (noncancerous cell line) in our experiments. Both cell lines were acquired from ATCC.

4T1 (ATCC CRL2539™) and HEK293T (ATCC CRL-3216) cell lines were cultured in DMEM High Glucose Culture Medium (BioSera) containing 10% FBS, 2 mmol/L glutamine, 100 U/mL penicillin, and 0.1 mg/mL streptomycin at 37°C. The medium was changed every 48 h and cells were passaged once weekly using standard trypsin-EDTA concentrations. Beginning at passage 32 and 37, respectively, cells were cultured continuously. Cells were frozen in freezing medium containing 10% FBS and 5% DMSO.

### 2.6. MTT Assay

The MTT tetrazolium salt is reduced by metabolically active cells, via the action of dehydrogenase enzymes. This leads to the generation of reducing equivalents such as nicotinamide adenine dinucleotide (NADH) and nicotinamide adenine dinucleotide phosphate (NADPH). The resulting intracellular purple formazan, from the initial yellow color of MTT, can be solubilized and quantified by spectrophotometry. The MTT cell viability assay measures alterations in cell viability thus; when metabolic events lead to apoptosis or necrosis, cell viability is decreased. As a general protocol, 5000 cells/well were seeded in 96-well plates (Corning-Costar, Corning, NY) and cultured overnight. Three different types of controls, namely, positive, negative, and background, were used throughout the study. Positive control had cells with culture medium but were not exposed to MNPs. Negative control had MNPs without cells. Background control had culture medium without cells. The two different cell lines were treated with various concentrations of MNPs for 24 h. Subsequently, the cells were rinsed once and incubated at 37°C with 100 *μ*l serum-free medium, containing 0.5 mg/mL MTT. After 1.5 to 2.5 h, 100 *μ*l of SDS-HCl was added to each well, mixed with the pipette and incubated for at least 1 h at 37°C. The optical densities were read at 570 nm (reference filter was set at 690 nm), using a microplate spectrophotometer (SPECTROstarNano, BMG LABTECH). Absorbances were normalized with respect to the untreated control cultures to calculate changes in cell viability.

### 2.7. Labeling of Fe_3_O_4_-DPD MNPs with ^68^Ga

For the radiolabeling experiment, ^68^Ga was eluted from the ^68^Ge/^68^Ga generator [33]. A fraction containing ^68^GaCl_3_ (~45 MBq) in a volume of 100 *μ*L was used. Radiolabeling was performed by mixing 50 *μ*L of Fe_3_O_4_-DPD NPs suspension (*C* = 3.2 mg/mL dispersed in water), 350 *μ*L of sodium acetate buffer (0.2 M, pH 4), and 100 *μ*L of ^68^GaCl_3_ and incubating at 90°C for 40 min. The radiolabeling yield was determined by thin layer chromatography analysis (TLC). The strip was developed using a 2 : 1 mixture of HCl/Acetone/deionized water and 2,4-Pentanedione as the mobile phase. With this system, ^68^Ga-Fe_3_O_4_-DPD MNPs remained at the application point, while unbound ^68^Ga^3+^ ions migrate with the solvent front. The radiolabeled sample was purified by centrifugation (12000 rpm, 10 min). After washing twice with deionized water, the supernatant was removed and the radiolabeled sample was redispersed in deionized water. The % radiochemical purity of ^68^Ga-Fe_3_O_4_-DPD MNPs was determined by TLC, as previously described. A control test was also carried out, in the absence of Fe_3_O_4_-DPD MNPs.

### 2.8. *In Vitro* Stability Studies of ^68^Ga-Fe_3_O_4_-DPD MNPs

To evaluate the* in vitro* stability of ^68^Ga-Fe_3_O_4_-DPD MNPs, a sample of 10 *μ*L ^68^Ga-Fe_3_O_4_-DPD MNPs was incubated with 90 *μ*L phosphate buffer saline (PBS) pH 7.4 while shaking at room temperature. For serum stability studies, 20 *μ*L ^68^Ga-Fe_3_O_4_-DPD MNPs were incubated with 180 *μ*L human serum at 37°C. The* in vitro* stability was determined at three time points (30, 60, and 120 min) by TLC, as described above.

### 2.9. Determination of the Size of ^68^Ga-Fe_3_O_4_-DPD MNPs

Atomic force microscopy (AFM) was used to assess the morphology and the size of ^68^Ga-Fe_3_O_4_-DPD MNPs. All measurements concerning the ^68^Ga-labeled MNPs were performed after the decay of ^68^Ga to the nonradioactive isotope ^68^Zn, in order to avoid any contamination due to the radioactive sample. Initially, the samples were spread onto cleaned standard microscope slides by means of an electronically rotating platform (rotation frequency 2000 rpm, rotation duration 10 sec) to prepare single layer films. Then, AFM images of the films were obtained by means of a scanning probe microscope [NT-MDT Solver PRO] set in noncontact tapping mode.

Dynamic light scattering (DLS) was also used to measure the size distributions of Fe_3_O_4_-DPD MNPs and ^68^Ga(→^68^Zn)-Fe_3_O_4_-DPD MNPs in aqueous solutions using a DLS apparatus. In a typical DLS measurement, 60 *μ*L of Fe_3_O_4_-DPD MNPs or 100 *μ*L of ^68^Ga(→^68^Zn)-Fe_3_O_4_-DPD MNPs diluted with 300 *μ*L ultrapure water was measured at 22°C. For each dispersion, at least ten light scattering measurements were collected and the results were averaged.

### 2.10. Magnetic Properties of Fe_3_O_4_-DPD and ^68^Ga-Fe_3_O_4_-DPD MNPs

The magnetic measurements of Fe_3_O_4_-DPD and ^68^Ga-Fe_3_O_4_-DPD samples were performed by means of a SQUID magnetometer (5.5 T MPMS, Quantum Design). As mentioned above, these measurements were performed after the decay of ^68^Ga to the stable isotope ^68^Zn. Each sample was measured at body temperature, *T* = 36°C, and in liquid form, dispersed in bidistilled water. To this end, we employed a plastic cylindrical container of appropriate diameter (~5 mm) that fits the opening of the SQUID sample chamber. The container was filled with 190 *μ*l MNPs (0.608 mg,* C* = 3.2 mg/ml) and sealed carefully. Magnetization measurements versus magnetic field, *M*(*H*), were performed both on the empty container and the sample-filled container under the exact same conditions. Thus, we were able to isolate the *M*(*H*) data of the Fe_3_O_4_-DPD and ^68^Ga(→^68^Zn)-Fe_3_O_4_-DPD MNPs samples by subtracting the signal of the empty container.

### 2.11. *Ex Vivo* Biodistribution Studies

Animal experiments were carried out according to European and national regulations. These studies have been further approved by the Ethics Committee of the NCSR “Demokritos” and animal care and procedures followed are in accordance with institutional guidelines and licenses issued by the Department of Agriculture and Veterinary Policies of the Prefecture of Attiki (Registration numbers: EL 25 BIO 022 and EL 25 BIO 021). Normal Swiss mice were obtained from the breeding facilities of the Institute of Biosciences and Applications, NCSR “Demokritos.” The animals were housed in air-conditioned rooms under a 12 h light/dark cycle and allowed free access to food and water.

The sample of ^68^Ga-Fe_3_O_4_-DPD MNPs was loaded onto a size exclusion PD-10 column, containing Sephadex G-25 resin and eluted with phosphate buffer saline (PBS), in order to eliminate aggregated ^68^Ga-Fe_3_O_4_-DPD MNPs, which may cause venous thrombosis during tail injections of the sample in the animals. Ten 0.5 ml fractions were collected, and the radioactivity of each fraction was measured using a dose calibrator (Capintec). The fractions containing the highest radioactivity were pooled and used for the study.

The* in vivo* behavior of ^68^Ga-Fe_3_O_4_-DPD MNPs was evaluated in 9 normal Swiss mice (weight 23–27 gr). Intravenous administration of 100 *μ*l PBS suspension (11.11 *μ*g/100 *μ*l ^68^Ga-Fe_3_O_4_-DPD MNPs per mouse) of purified ^68^Ga-Fe_3_O_4_-DPD MNPs was performed via the tail vein. The animals were sacrificed at 30, 60, and 120 min postinjection (3 mice per time-point). Then, samples of blood and organs were excised, weighed, and measured for radioactivity in a Gamma scintillation counter. The remaining radioactivity in the tail, as well as background counts was subtracted, and the radioactivity decay was autocorrected by the counter. Then, the accumulation of ^68^Ga-Fe_3_O_4_-DPD MNPs in each organ was expressed as the percentage injected activity per gram of tissue (% IA/g ± SD) and calculated compared to the activities of a standard dose of the injected solution.

### 2.12. *In Vivo* Imaging Studies

Dynamic coincidence images of the administered mice were obtained from 10 min up to 60 min p.i. Successive 2 min frames were collected, showing the gradual biodistribution of the studied substance. Frames were also summed, to achieve cumulative images of higher statistics. All functional images were planar, coincidence images and no tomographic reconstruction was performed. Upon completion of the coincidence imaging, X-ray images were also acquired at the exact same mouse positioning to act as an anatomical guide for the organs' exact location. The X-ray imaging parameters were set to 35 kVp, 500 *μ*A, and 0.1 s exposure time. Fusion between coincidence and X-ray images was performed semiautomatically through an in-house standard procedure. The two heads were not rotated and no tomographic imaging was performed.

The images were stored in raw format and postprocessed with ImageJ open source software (version 1.49v; NIH). Then, they were linearly interpolated, to provide a smoother final image. No smoothing algorithm was used. ImageJ was also used to select the color map and enhance image contrast at a certain level where the organs/structures of interest can be distinguished.

## 3. Results and Discussion

In the present study, Fe_3_O_4_ nanoparticles have been coated with DPD. The primary idea behind the design and synthesis of these MNPs was to improve stability and biocompatibility, as well as provide a platform for the development of a theranostic agent. As was recently shown in a study by Djokić et al. [29], MNPs coated with DPD efficiently complexed Yttrium-90, leading to a radiolabeled species for therapeutic applications. Furthermore, DPD offers the capability to radiolabel the same MNPs with ^68^Ga, for diagnostic applications.

### 3.1. Synthesis and Characterization of the Fe_3_O_4_-DPD MNPs

**Figure 1:**
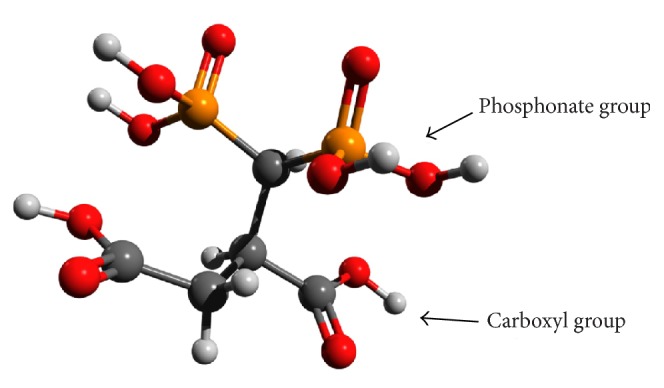
DPD ligand.

In this study, MNPs were synthesized according to the coprecipitation method and stabilized with the tetradentate ligand DPD (Figure 1). The surface of MNPs was modified with hydrophilic carboxylate and phosphonate groups shortly after the particle formation, resulting in increased stability and dispersibility of the carrier in aqueous solution.

**Figure 2:**
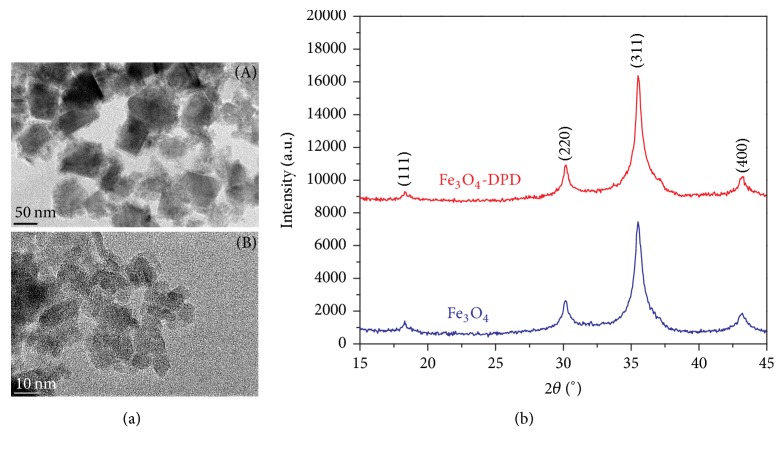
((a), (A) and (B)) TEM images of DPD-coated MNPs; (b) XRD patterns of Fe_3_O_4_ and Fe_3_O_4_-DPD MNPs.

Transmission electron microscopy (TEM) was used in order to determine particle size and the distribution and morphology of Fe_3_O_4_-DPD MNPs (Figure 2(a), (A) and (B)). From TEM micrographs, it is observed that the MNPs were pseudospherical or discrete squares with a diameter of around 6 nm. Particles were agglomerated due to magnetic interactions among them.

Crystal structures of the MNPs were checked by X-ray powder diffraction method. In the obtained diffraction patterns (Figure 2(b)) all reflections were indexed in the expected space group,* Fd*$\overset{-}{3}$*m*, and spinel structure type. The mean crystallite diameter of 5.6 nm was estimated for the Fe_3_O_4_ using Scherer's equation and the peak half-height width of the (311) reflections. The same values were obtained using XRD data for pure Fe_3_O_4_ and Fe_3_O_4_-DPD MNPs. The mean crystallite size calculated from the XRD patterns is consistent with particle size estimated from the relevant TEM micrographs. Hence, particles are composed from one crystallite.

**Figure 3:**
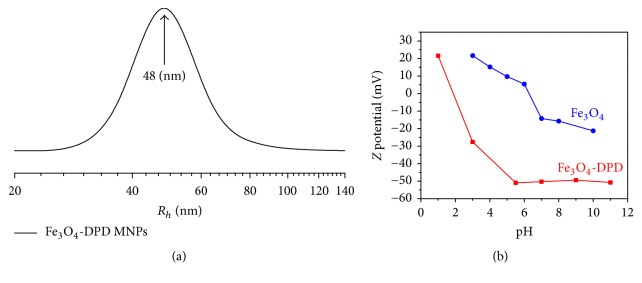
(a) Intensity weighted hydrodynamic radii size distribution of Fe_3_O_4_-DPD MNPs; (b) dependence of zeta potential of Fe_3_O_4_ and Fe_3_O_4_-DPD MNPs on the change of pH.

DLS measurements were carried out on the Fe_3_O_4_-DPD MNPs (Figure 3(a)). The sample was diluted with ultrapure water and measured at 22°C. A sharp monomodal size distribution of the MNPs was observed. Using CONTIN analysis of DLS measurements, it was found that the mean size distribution of intensity weighted hydrodynamic diameter of Fe_3_O_4_-DPD MNPs was 96 nm. The polydispersity index (PDI) of Fe_3_O_4_-DPD MNPs was calculated from the cumulants analysis and its value was less than 0.3, indicating that the nanoparticles have a considerably narrow size distribution and are essentially monodispersive. As PDI values are very sensitive to the presence of aggregates, it is obvious that the Fe_3_O_4_-DPD MNPs are stable since PDI is not affected over time (Table 1). It should be noticed that the results obtained from DLS and TEM are different since the first obtained direct from solution and measured the hydrodynamic radii of the particles, while the second obtained from dried solution where the particles aggregated due to the removal of water. Zeta potential measurements as a function of pH for the Fe_3_O_4_ and Fe_3_O_4_-DPD MNPs are shown in (Figure 3(b)). Due to the presence of carboxylate and phosphonate groups on the surface of Fe_3_O_4_-DPD MNPs, the surface charge of the MNPs was observed to be highly negative (−50.4 mV at pH = 7) and shows high electrostatic repulsions between the charged nanoparticles, which ensures the colloidal stability. Again, this confirms the efficiency of coating since the IEP (1.9) has been shifted to lower pH values compared to naked MNPs (6.2). The correlation of zeta potential to pH is important to know so that one can predict how the pH inside the human body will affect the surface charge of the nanoparticles and therefore protein adsorption onto nanoparticles. Fe_3_O_4_-DPD MNPs showed high stability in aqueous medium as their size and zeta potential remained unaffected over a long period of time (Table 1).

**Figure 4:**
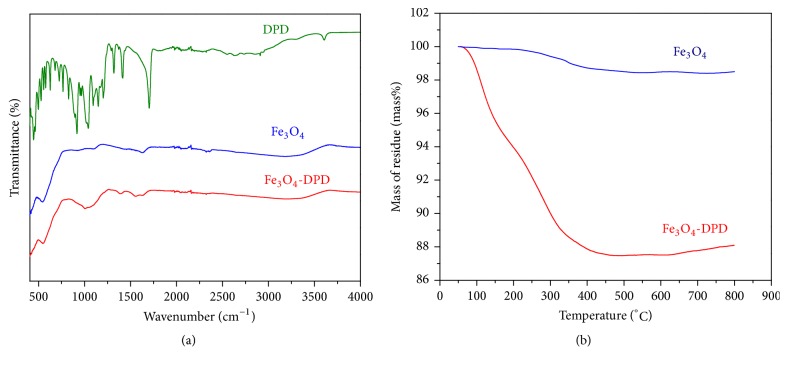
(a) ATR-FTIR spectra of DPD, Fe_3_O_4_, and Fe_3_O_4_-DPD MNPs; (b) TGA curves of Fe_3_O_4_ and Fe_3_O_4_-DPD MNPs.

Further conformation of effective coating of the DPD ligand on the surface of MNPs came from FTIR spectroscopy and thermogravimetric analysis. The infrared spectra of the free ligand DPD, Fe_3_O_4_ MNPs, and Fe_3_O_4_-DPD MNPs are shown for comparison (Figure 4(a)). DPD could coordinate the metal through the carboxylate and the phosphonate groups. In that case significant changes for the bands of the carboxylate and the phosphonate groups of the Fe_3_O_4_-DPD complex should be seen in its infrared spectra. The most important differences between DPD and Fe_3_O_4_-DPD spectra are related to the phosphonate bands in the region 1200–800 cm^−1^.

Comparing the Fe_3_O_4_-DPD MNPs (red curve) with the free DPD ligand (green curve) (Figure 4(a)), the large changes observed within the P–O stretching region (1200–900 cm^−1^) show that a strong interaction between the phosphonate group and the Fe_3_O_4_ is present. The spectrum of the free DPD exhibits, within the P–O stretching region (1200–900 cm^−1^), two sharp peaks at 1204 and 918 cm^−1^, assigned to P=O and P–OH, respectively. The broadband at 1040 cm^−1^ in the spectrum of Fe_3_O_4_-DPD MNPs is characteristic for the vibrational mode for the PO_3_^2−^ group. Appearance of this broad phosphoryl band, as well as the disappearance of the P = O and P–O–H bands, indicates a mainly tridentate binding of DPD to the surface of Fe_3_O_4_ MNPs. For the DPD ligand, the carboxylate ions present two characteristic bands at 1704 and 1414 cm^−1^ due to the asymmetric and symmetric carboxylate stretches, respectively. Comparing the Fe_3_O_4_-DPD MNPs with the free DPD, the characteristic carboxylate stretches were shifted to lower frequencies at 1635 and 1396 cm^−1^, respectively, which suggests that DPD molecules are bound to the particle surface also via –COO^−^ groups. The ATR-FTIR spectrum of Fe_3_O_4_-DPD MNPs exhibited a strong band at 540 cm^−1^, characteristic of the Fe-O vibration related to the magnetite core. Thermogravimetric analysis (TGA) of naked MNPs detected no significant peaks (Figure 4(b)). There was a 3% weight loss as the temperature increased from 100°C to 800°C, which might be due to a loss of absorbed water. In contrast, the functionalized MNPs lost weight in two steps. The first step occurred over the range 25–200°C and might also be due to the loss of absorbed water. The second step consisted of a weight loss of about 12.5% over the range 200–450°C and might be due to the burning of bonded ionic liquids. At higher temperatures of 450–900°C, the weight remained constant, implying the presence of only Fe_3_O_4_ left within the temperature range.

**Figure 5:**
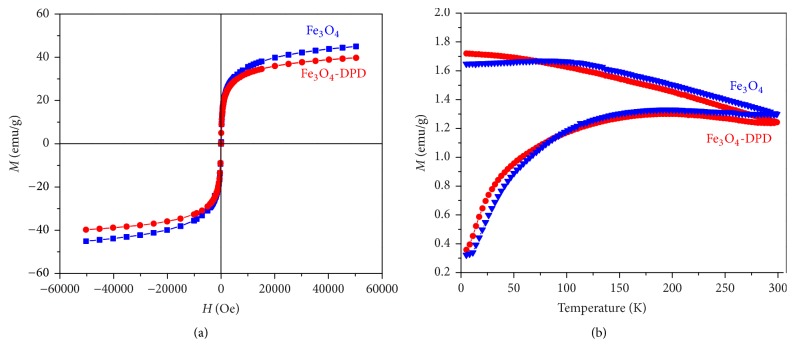
(a) Hysteresis loop for Fe_3_O_4_ and Fe_3_O_4_-DPD MNPs at 300 K; (b) temperature dependence on magnetization of the Fe_3_O_4_ and Fe_3_O_4_-DPD MNPs taken in zero-field (lower branch) and field-cooling (upper branch) modes.

Magnetic properties of Fe_3_O_4_ and Fe_3_O_4_-DPD MNPs were investigated by magnetization measurements versus field, *M*(*H*) at 290 K as well as by ZFC-FC magnetization from 2–300 K in applied field of 100 Oe. Magnetization versus field, *M*(*H*), curves are shown in Figure 5(a). It can be observed that the magnetization does not fully saturate up to 50 kOe, reflecting the hard magnetic behavior of the particle surface. The saturation magnetization,* M*_*s*_, as determined by extrapolating *M*(1/*H*) to* H* = 0, was* M*_*s*_ = 47 and 42 emu/g, for Fe_3_O_4_ and Fe_3_O_4_-DPD, respectively. Remanent magnetization, *M*_*R*_, and coercivity, *H*_*C*_, values are nearly zero, in agreement with the superparamagnetic behavior expected for these nanoparticles on the time scale of magnetization measurement time *τ*_*M*_ ~ 100 s. Temperature dependencies of ZFC and FC magnetization for Fe_3_O_4_ and Fe_3_O_4_-DPD MNPs are shown in Figure 5(b). The ZFC and FC magnetization curves furcate at the maximum temperature of measurements, 300 K. A broad maximum in ZFC branch around 200 K is a consequence of a broad particle size distribution, as was shown by TEM examinations (Figure 2(a), (B)). In the FC branches magnetization increases a little bit from room temperature to 100 K, and below it shows a tendency to be saturated. The found FC behavior indicates the interparticle interactions and probably a spins glass state.

### 3.2. *In Vitro* Cytotoxicity Study of Fe_3_O_4_-DPD MNPs

Although the chemical characterization of the synthesized Fe_3_O_4_-DPD MNPs is crucial in order to further characterize their diagnostic utility as a PET radiotracer, quantitative assays of the metabolic activity of cancer cell lines could grant a better knowledge of the mechanisms implied in the toxicity caused by those MNPs. The assays based on the measurement of the metabolic activity of the cells are the most common methods in order to designate the cell viability under nanoparticle treatment. The MTT viability assay intends to track the activity of reductase enzymes in order to measure the cell viability of 4T1 and HEK293 cell lines and thus the cytotoxicity caused by treatment with various concentrations of Fe_3_O_4_-DPD MNPs (3, 5, 10, 20, 25, and 30 *μ*g/ml) for 24 h, an appropriate incubation time for Fe_3_O_4_-DPD MNPs internalization from the cells [34].

**Figure 6:**
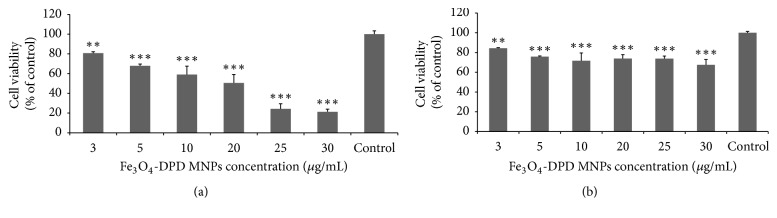
MTT cell viability assay after 24 h treatment of (a) 4T1 cells and (b) HEK293T cells with Fe_3_O_4_-DPD MNPs. Positive control shows cells without exposure to MNPs. Cell viability is expressed as % cell viability ± SD between two experiments. The symbols *∗∗* and *∗∗∗* show statistical significance using one-way ANOVA (*p* < 0.01 and *p* < 0.005, resp.), compared to positive control.

According to our results, the viability of cancer 4T1 cells shows a 50% reduction (*p* < 0.005) when treated with 20 *μ*g/ml, while the viability of the cells falls to 20% under treatment with 30 *μ*g/ml (*p* < 0.005) of Fe_3_O_4_-DPD MNPs (Figure 6(a)). Thus, the cytotoxicity results demonstrate a dose-dependent cytotoxicity effect of Fe_3_O_4_-DPD MNPs in 4T1 cancer cell line. Recently, several* in vitro *experiments in eukaryotic cells show that internalized MNPs, depending on their size and modification, can induce mitochondrial dysfunction, increase the level of ROS, and subsequently cause DNA damage, chromosomal aberrations, apoptosis, impairment of the cell membrane, and cell cycle rest. The later effects are probably caused by the ions released (ROS generation) by magnetic MNPs that lead to the ignition of oxidative stress responses into the cell and at the end to cytotoxicity. Moreover, although iron MNPs degraded in the cell, they can change the cellular iron pool, leading to aberrant expression of transferrin receptor, cyclins, and induction of apoptotic responses [35–37]. Regarding cytotoxicity, the choice of the cell line tested under MNPs treatment is of great importance, since it is demonstrated that MNPs toxicity is highly cell-type dependent [38, 39]. Therefore, the cytotoxicity of Fe_3_O_4_-DPD MNPs against a noncancerous cell line (HEK293) was also tested and it was shown that toxicity against this cell line is greatly reduced, compared to the 4T1 cell line (Figure 6(b)). Even at a concentration of 30 *μ*g/ml the viability is >60% (*p* < 0.005). These results indicate selective cytotoxic effects of Fe_3_O_4_-DPD MNPs in cancer cells with regard to normal cells. Further experiments are needed in order to elucidate the mechanism by which Fe_3_O_4_-DPD MNPs cause higher cytotoxicity in cancer cells, thus highlighting their potential use as therapeutic agents.

### 3.3. Radiolabeling of Fe_3_O_4_-DPD MNPs with ^68^Ga

Fe_3_O_4_-DPD MNPs were radiolabeled with ^68^Ga radionuclide without the presence of a chelator. In our study DPD, a tetradentate ligand with two phosphonates and two carboxylates serves as an effective ligand that coordinates with ^68^Ga^3+^. The radiolabeling yield of the sample was found to be ~70%, as determined by radio-TLC analysis. Moreover, the radiolabeled sample was purified via centrifugation and afforded a radiochemical purity of >91%. The radiolabeling results are in agreement with the literature [21, 40–44].

### 3.4. *In Vitro* Stability Studies of ^68^Ga-Fe_3_O_4_-DPD MNPs

A significant factor to be considered when developing a new radiolabeled nanoparticle is that the radionuclide must be bound to the nanoparticle to form a stable conjugate under physiological conditions to avoid their separation and nonspecific deposition of free ions in tissues. Otherwise, biodistribution and imaging data will not indicate the fate of nanoparticles, as the radionuclide distribution will not reflect that of the nanoparticles.

With the aim of assessing the* in vitro* stability of ^68^Ga-Fe_3_O_4_-DPD MNPs in biological media, the radiolabeled sample was incubated with PBS and human serum. The results exhibited satisfactory* in vitro* stability in PBS (~80% stable ^68^Ga-Fe_3_O_4_-DPD MNPs) and high* in vitro* stability in human serum (>92% stable ^68^Ga-Fe_3_O_4_-DPD MNPs), as evaluated by TLC analysis, at three time points (30, 60, and 120 min) after incubation. Our results are in accordance with work reported by other groups [18, 21, 26, 28, 40, 41].

### 3.5. Determination of the Morphology and Size of  ^68^Ga-Fe_3_O_4_-DPD MNPs

The size of radiolabeled magnetic nanoparticles highly affects their pharmacokinetics and* in vivo* behavior. Nanoparticles larger than 200 nm are sequestered by macrophages of liver and spleen, while nanoparticles smaller than 10 nm are rapidly removed through renal clearance. Also, nanoparticles bigger than 4 *μ*m in diameter are mainly captured in the lungs and may lead to the risk of embolism. Thus, nanoparticles with a mean size in the range of 10–200 nm escape opsonization exhibit longer blood circulation times and are most effective for biological applications. In our study, AFM measurements were performed to assess the size of the ^68^Ga-Fe_3_O_4_-DPD MNPs as described elsewhere [45]. These measurements were performed after the decay of ^68^Ga to the stable isotope ^68^Zn, as mentioned in Materials and Methods.

**Figure 7:**
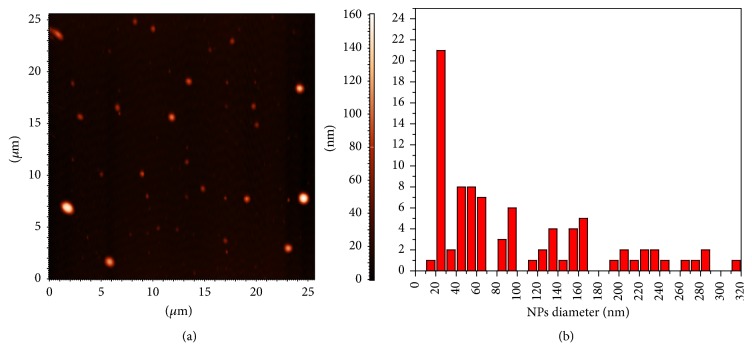
(a) Typical AFM image showing the morphology of ^68^Ga(→^68^Zn)-Fe_3_O_4_-DPD MNPs; (b) size distribution of ^68^Ga(→^68^Zn)-Fe_3_O_4_-DPD MNPs measured from several AFM images.

The acquired AFM images showed that the size of ^68^Ga(→^68^Zn)-Fe_3_O_4_-DPD MNPs ranged from 30 to 180 nm (*N* = 87 nanoparticles measured from several AFM images). As shown in Figure 7(a) the nanoparticles are homogenous and spherical, while the majority of the nanoparticles are less than 50 nm (Figure 7(b)). Some aggregates of varying sizes may be created in the radiolabeled sample during the radiolabeling process. It has also been reported that PEG-coated superparamagnetic iron oxide nanoparticles subjected to high salt concentration (0.2 M ammonium acetate buffer) during radiolabeling procedure with ^68^Ga exhibit larger size compared to their size before the radiolabeling [27].

**Figure 8:**
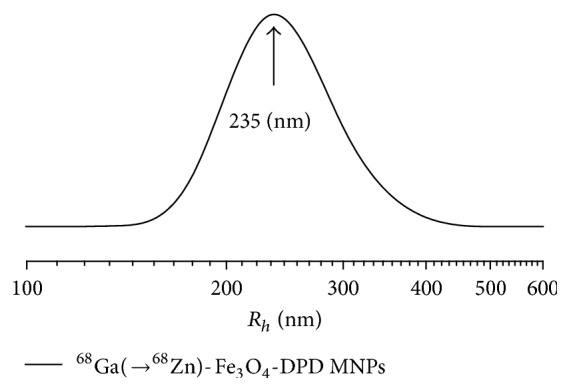
Intensity weighted hydrodynamic radii size distribution of ^68^Ga(→^68^Zn)-Fe_3_O_4_-DPD MNPs.

DLS measurements were also carried out on the radiolabeled sample following the procedure described above. Also in this case, a sharp monomodal size distribution of ^68^Ga(→^68^Zn)-Fe_3_O_4_-DPD MNPs with average intensity weighted hydrodynamic diameters of about 400–500 nm was observed (Figure 8). The value of mean diameter is higher than that of the nonradiolabeled sample due to the radiolabeling process as mentioned before.

### 3.6. Magnetic Properties of Fe_3_O_4_-DPD and ^68^Ga(→^68^Zn)-Fe_3_O_4_-DPD MNPs

**Figure 9:**
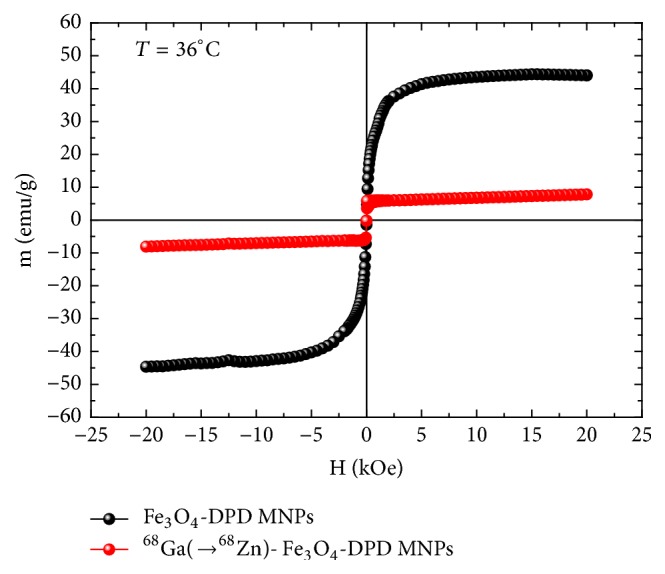
Magnetization versus magnetic field at* T* = 36°C for Fe_3_O_4_-DPD and ^68^Ga(→^68^Zn)-Fe_3_O_4_-DPD MNPs. Both samples were in liquid form, dispersed in bidistilled water.

Figure 9 shows representative magnetization measurements of Fe_3_O_4_-DPD and ^68^Ga(→^68^Zn)-Fe_3_O_4_-DPD MNPs as they were measured by means of a SQUID magnetometer at conditions of body temperature (*T* = 36°C) and in liquid form (dispersed in bidistilled water) since we wanted to simulate, at least at the laboratory level, the conditions that these conjugates will ultimately be used in subsequent studies on animal models. As shown in this figure, the saturation magnetization of Fe_3_O_4_-DPD MNPs is* M*_*s*_ = 44 emu/g, while that of ^68^Ga(→^68^Zn)-Fe_3_O_4_-DPD MNPs is* M*_*s*_ = 8 emu/g. The observed reduction of* M*_*s*_ is attributed to two unavoidable factors: first, the reduction of the effective concentration of the Fe_3_O_4_ content during the radiolabeling process of the Fe_3_O_4_-DPD MNPs sample (mass loss of Fe_3_O_4_ during centrifugation/washing cycles needed to remove free ^68^Ga that possibly exists in the supernatant and inability to completely remove all supernatant before the final dilution to the specified final liquid volume); second, to the change in the chemical composition of Fe_3_O_4_ to Fe_2_O_3_ due to oxidization (the radiolabeling process is performed in atmosphere, at relatively high temperature conditions,* T* = 90°C). Finally, we should note that neither the Fe_3_O_4_-DPD nor the ^68^Ga(→^68^Zn)-Fe_3_O_4_-DPD MNPs were aggregated even under the application of a magnetic field as high as 20 kOe.

### 3.7. *Ex Vivo* Biodistribution Studies

**Figure 10:**
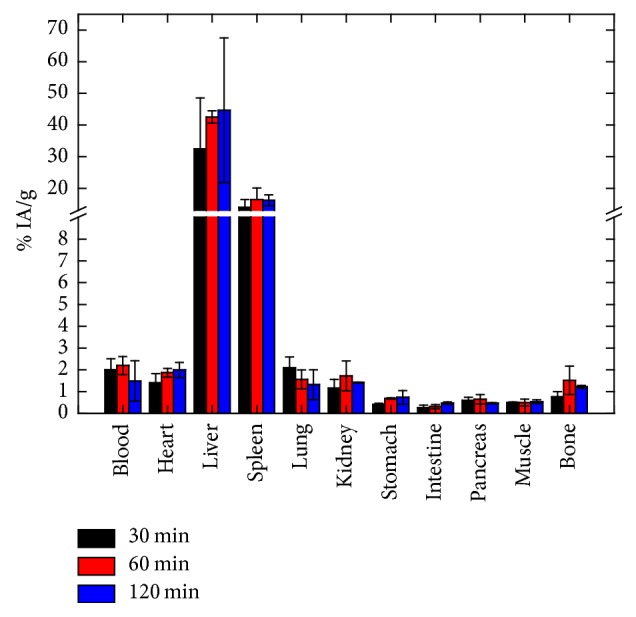
*Ex vivo* biodistribution study of ^68^Ga-Fe_3_O_4_-DPD MNPs in normal Swiss mice performed at 30, 60, and 120 min postinjection.

The* ex vivo* biodistribution of ^68^Ga-Fe_3_O_4_-DPD MNPs was performed to assess their* in vivo* behavior as potential PET/MRI imaging agents. The ^68^Ga-Fe_3_O_4_-DPD MNPs were administered via tail vein injection in normal Swiss mice. In general, the accumulation of ^68^Ga-Fe_3_O_4_-DPD MNPs in the organs at all time points examined is presented in Figure 10, as percentage of injected activity per gram tissue (% IA/gr ± SD). ^68^Ga-Fe_3_O_4_-DPD MNPs were distributed throughout the organs, while in liver and spleen the uptake was the highest. Specifically, the blood retention of the nanoparticles was 2.19 ± 0.42% IA/g up to 60 min postinjection (p.i.) and showed a decrease to 1.49 ± 0.92% IA/g at 120 min p.i. The accumulation of ^68^Ga-Fe_3_O_4_-DPD MNPs in the heart was increased with time (1.41 ± 0.42% IA/g at 30 min, 1.87 ± 0.20% IA/g at 60 min, and 1.99 ± 0.35% IA/g at 120 min p.i.). On the contrary, in the lungs the uptake showed opposite behavior (2.10 ± 0.49% IA/g at 30 min, 1.57 ± 0.43% IA/g at 60 min, and 1.32 ± 0.69% IA/g at 120 min p.i.). The small percentage of the uptake in lungs can be attributed to possible embolization caused by postinjection aggregation.

The organs of the reticuloendothelial system (RES) exhibit the highest accumulation of ^68^Ga-Fe_3_O_4_-DPD MNPs, compared to the other organs. In the liver, there were 32.42 ± 16.12, 42.52 ± 1.95, and 44.66 ± 22.86% IA/g at 30, 60, and 120 min p.i., while in the spleen the uptake reached the 16.48 ± 3.58% IA/g at 60 min p.i. and remained relatively constant (16.21 ± 1.74% IA/g) up to 120 min p.i. These organs are rich in macrophages (i.e., liver's Kupffer cells), which are able to recognize and engulf ^68^Ga-Fe_3_O_4_-DPD MNPs through phagocytosis. The kidney uptake was relatively low at all time points. All other organs studied showed low or negligible uptake.

It is well known that following intravenous injection of radiolabeled magnetic nanoparticles in the bloodstream, plasma proteins, namely, opsonins, are adsorbed onto the surface of the nanoparticles [46]. These plasma proteins are recognized by the macrophages mostly found in the liver and spleen, resulting in rapid clearance of the nanoparticles from the bloodstream. The opsonization process and therefore the* in vivo* behavior of the radiolabeled magnetic nanoparticles are highly affected by their physicochemical properties, such as their coating, size and surface charge.

Despite the coating with DPD, a biocompatible and hydrophilic coating, the ^68^Ga-Fe_3_O_4_-DPD MNPs displayed long-term retention in the liver, as discussed above. This behavior was similar to ^90^Y-labeled Fe_3_O_4_ MNPs coated with PEG600 diacid, with a size of 46 ± 0.6 nm [18]. This behavior may be attributed to the size of the ^68^Ga-Fe_3_O_4_-DPD MNPs as measured with AFM [46, 47]. As discussed by Chouly et al. [48], a similar behavior was observed for ^59^Fe-labeled dextran-coated Fe_3_O_4_ MNPs with different diameters (30 nm–100 nm). When injected in Swiss mice, they showed decreased blood retention with increasing size, while the liver accumulation was increased following the increasing size of the nanoparticles.

Another fact to be considered is the surface charge of Fe_3_O_4_-DPD MNPs. It has been shown that a neutral charge interacts minimally with the plasma proteins and thus contributes to the extended circulation time of the MNPs, whereas a high surface charge enhances the phagocytosis process [46]. Positively charged nanoparticles show nonspecific internalization rate, plasma protein binding, aggregation, and short blood circulation half-life [47]. In a study carried out using dextran-coated Fe_3_O_4_ MNPs with different surface charges (neutral, negative −30 mV, positive +20 mV) [48], the accumulation of nanoparticles in the liver was 3 times lower for the neutral nanoparticles than the charged ones, while the negatively charged nanoparticles exhibited increased liver uptake. In our study, the highly negative surface charge of Fe_3_O_4_-DPD MNPs (−50.4 mV at pH = 7) led to high electrostatic repulsions between the charged nanoparticles, thus ensuring the colloidal stability of the nanoparticles, while their high liver uptake can be attributed to their negative surface charge.

### 3.8. *In Vivo* Imaging Studies

**Figure 11:**
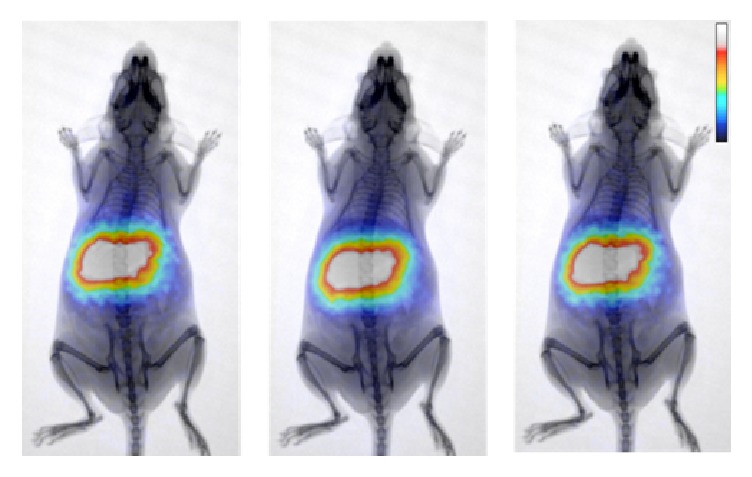
Cumulative PET/X-ray images of a normal Swiss mouse injected with ^68^Ga-Fe_3_O_4_-DPD MNPs at 20, 30, and 60 min p.i. The gradual alteration in color indicates a lower to higher number of recorded counts.

Dynamic and cumulative imaging studies of anesthetized healthy Swiss mice were performed on a dedicated small animal PET/X-ray system, up to 1 hour p.i. (Figure 11). Imaging and biodistribution studies were found to be in good agreement. Kidney uptake is not discernable in the PET images, due to the significant concentration in the liver and the partial overlapping of kidneys with liver and intestines.

## 4. Conclusions

Iron oxide nanoparticles were successfully synthesized and surface functionalized with the biocompatible and water soluble stabilizer DPD, providing the bare Fe_3_O_4_ MNPs with the appropriate dispersing stability, minimizing their potential cytotoxicity and exhibiting chelating properties. Fe_3_O_4_-DPD MNPs showed reduced toxicity in the normal cells, compared to cancer cells and efficient labeling with the positron emitter Ga-68 to form stable constructs. Although* ex vivo* biodistribution and* in vivo* PET imaging studies of ^68^Ga-Fe_3_O_4_-DPD MNPs showed high accumulation in the RES organs, they indicated satisfactory blood retention at 30, 60, and 120 min p.i. As a result, ^68^Ga-Fe_3_O_4_-DPD MNPs exhibit great potential as a PET/MR imaging agent. We are currently working on the assessment of ^68^Ga-Fe_3_O_4_-DPD MNPs in tumor bearing animal models to evaluate their application in cancer imaging.

## Figures and Tables

**Table 1:** Stability analysis of Fe_3_O_4_-DPD MNPs, variation in PDI and zeta potential against time.

Time (months)	1	2	3	4	5	6
PDI	0.255	0.241	0.267	0.287	0.263	0.271
Zeta potential (mV)	−49.8	−50.1	−48.6	−49.2	−50.3	−50.4
